# Metaplastic breast carcinoma development following surgical resection of an inflammatory myofibroblastic tumor in the right breast: A case report

**DOI:** 10.3892/ol.2014.2261

**Published:** 2014-06-17

**Authors:** PENG XING, JIGUANG LI, FENG JIN, YUNFEI WU, XINYU ZHENG, BO CHEN, FAN YAO, XIAOLIN WEI

**Affiliations:** Department of Breast Surgery, The First Affiliated Hospital of China Medical University, Shenyang, Liaoning 110001, P.R. China

**Keywords:** benign disease, breast carcinoma, case report, malignant progression

## Abstract

Inflammatory myofibroblastic tumors (IMTs) are uncommon, mesenchymal lesions, and malignant transformation is extremely rare. The current study presents the case of a 56-year-old female with a rapidly growing mass in the right breast, which was diagnosed as IMT. Immunohistochemically, the mass was positive for smooth muscle actin (SMA) and Ki-67 (positive staining in 30% of the cells), and negative for S-100, cluster of differentiation (CD)34, p63 and cytokeratin. Malignant transformation to metaplastic carcinoma of the spindle-cell type was observed following surgical resection. This metaplastic carcinoma demonstrated positive immunoreactivity for cytokeratin, vimentin, CD34, p63 and Ki-67 (>30%), and was negative for cytokeratin 7, SMA, desmin and S-100. The patient underwent total mastectomy of the right breast, followed by palliative chemotherapy with capecitabine; however, the patient succumbed to the disease after 12 weeks. The unusual case presented in the current study emphasizes the importance of pre-operative examinations to determine the benign or malignant nature of IMTs, which aids in the choice of appropriate surgical procedures.

## Introduction

Inflammatory myofibroblastic tumors (IMTs) are uncommon, mesenchymal neoplasms that are composed of proliferative myofibroblasts and infiltrating inflammatory cells, usually plasma cells and lymphocytes (1). The tumors have been documented to occur in various anatomical sites, including the lungs, abdomen, retroperitoneum, pelvis, trunk, peripheral nerves, soft tissue and breast (2,3). The etiology of IMT remains uncertain and controversial. IMT is generally accepted as a benign disease, however, in certain cases, a malignant phenotype is observed. For example, Carillo *et al* (4) reported a case of a bilateral pulmonary IMT with left adrenal gland metastasis. Complete surgical excision, when feasible, remains the primary treatment of choice for IMT (5). For inoperable cases, however, a treatment regimen with chemotherapy or radiotherapy is adopted (6). The current study presents an unusual case of IMT of the breast, with malignant transformation to a metaplastic carcinoma following surgical resection. Consent was obtained from the patient’s relatives.

## Case report

A 56-year-old female was admitted to a local hospital for the treatment of a rapidly growing mass in the right breast. The patient claimed that the mass had been present for >20 years, but had profoundly increased in size during the two months prior to admission. A computed tomography (CT) scan confirmed a solitary mass, ~9×6 cm in size, with a clear margin. Subsequently, the patient underwent lumpectomy of the mass. Based on the post-operative histopathological examination, the mass was diagnosed as IMT, with positive staining for smooth muscle actin (SMA) and Ki-67 (intense nuclear staining in 30% of the cells), and negative staining for S-100, cluster of differentiation (CD)34, p63 and cytokeratin.

**Figure 1:**
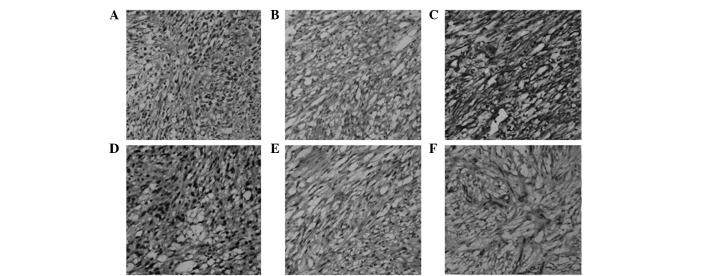
(A) Histopathological findings prior to total mastectomy. Hematoxylin and eosin staining analysis revealed that the tumor consisted of spindle-shaped cells (myofibroblasts and fibroblasts) arranged loosely and admixed with inflammatory infiltrates. Immunohistochemically, the tumor was negative for (B) cytokeratin and positive for (C) vimentin, (D) Ki-67, (E) p63 and (F) cluster of differentiation (CD)34. Magnification, ×200.

The patient suffered a relapse two months after the lumpectomy and was transferred to the Department of Breast Surgery, the First Affiliated Hospital of China Medical University (Shenyang, Liaoning, China). Enhanced CT and ultrasonography examinations revealed no abnormalities in the lungs, liver, gallbladder or spleen. However, the patient exhibited a low serum hemoglobin level of 57 g/l (normal range, 110–150 g/l). The recurrent tumor mass was growing progressively and ulceration was evident on the surface. Histologically, the tumor was predominantly composed of a conspicuous proliferation of spindle cells arranged in a loose fashion and surrounded by infiltrating inflammatory cells. In addition, partial necrosis was noted (<10%). Immunohistological studies revealed that the tumor yielded positive staining for p63, vimentin, CD34 and CD68, but was negative for SMA, CD38 and cytokeratin (Fig. 1). Positive Ki-67 staining was observed in >40% of the cells (Fig. 1), and five satellite lesions were identified, with a mean diameter ranging between 1 and 2 cm. These clinical and pathological findings confirmed the diagnosis of IMT with malignant transformation.

**Figure 2:**
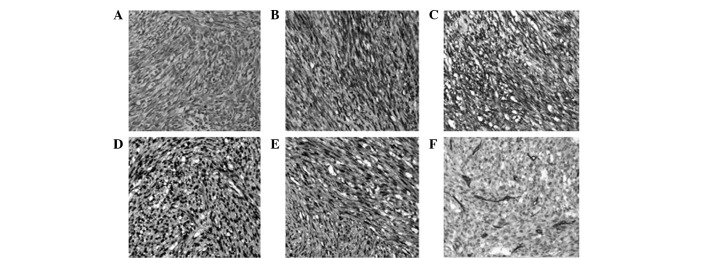
(A) Histopathological findings following total mastectomy. Hematoxylin and eosin staining analysis revealed that the recurrent tumor was predominantly composed of scattered spindle cells with atypical mitotic features. Immohistochemically, the tumor was positive for (B) cytokeratin, (C) vimentin, (D) Ki-67, (E) p63 and (F) cluster of differentiation (CD)34. Magnification, ×200.

With regard to treating the symptomatic anemia, 10 units of leukocyte-filtered red blood cells were administered prior to surgery. The patient then underwent a total mastectomy of the right breast. During the surgery, the tumor (16×15×15 cm in size) was found to intrude into the junctions of the ribs (third and fourth) and into the sternum. The final pathology revealed metaplastic carcinoma of the breast, predominantly composed of scattered spindle cells with atypical mitotic features. The immunoreactivity for the carcinoma was positive for cytokeratin, vimentin, CD34, p63 and Ki-67 (>30%), and negative for cytokeratin 7, SMA, desmin and S-100 (Fig. 2). Pathological examination indicated the presence of invading cancer cells in all three resected axillary lymph nodes. At 16 days post-surgery, local recurrence was observed in the right chest wall, coupled with the emergence of three satellite lesions (~1 cm in diameter). Due to the inoperable nature of the disease, the patient was referred to the Department of Oncology for palliative chemotherapy with capecitabine (2.5 g/m^2^ twice daily for 30 days) but succumbed to the disease after 12 weeks.

## Discussion

IMT has been detected in multiple locations, including the lungs, abdomen, pelvis, trunk, peripheral nerves and soft tissue (2,3). IMT of the breast, however, is an extremely rare entity and only a few cases have been reported in the English language literature (7,8). No specific signs or symptoms have been associated with IMT, and the exact diagnosis is usually based on pathological and immunohistochemical findings following resection of the tumor. The neoplastic nature of IMT (benign or malignant) remains a subject of debate. Idrees *et al* (9) reported two cases of benign laryngeal IMTs that appeared clinically as large infiltrating masses. Similarly, Ezzine-Baccari *et al* (10) described a benign pulmonary IMT with locally aggressive behavior. However, local recurrence and distant metastases are also encountered in certain cases of IMT (4). Due to the tendency towards local recurrence and the small risk of distant metastasis, IMT is classified as a tumor of intermediate biological potential by the World Health Organization (11). Rapid tumor growth and a high Ki-67 labeling index are associated with the aggressive behavior. The small tumor size (<3 cm) is regarded as a favorable prognostic factor for overall survival in patients with pulmonary IMT (12). In the present case, the tumor mass rapidly enlarged in size and a high Ki-67 labeling index was observed (30–40%). The tumor was positive for CD68, a macrophage-specific marker, indicating a pronounced infiltration of macrophages; accumulating evidence has linked tumor-associated macrophages and tumor progression (13). These findings indicate an aggressive potential of the IMT.

Although relatively rare, several cases of IMT with malignant progression have been reported. For example, Carillo *et al* (4) described a case of IMT of the bladder with rapid malignant progression, where multiple lymph node, bone and soft tissue metastases were observed on positron emission tomography. Malignant transformation of the IMT was also observed. Local recurrence with axillary lymph node metastases occurred even after total mastectomy. Histologically, the recurrent tumor mass was consistent with a metaplastic carcinoma, which was predominantly composed of spindle cells with atypical mitotic features. Furthermore, the immunostaining findings demonstrated that such malignant transformation was accompanied by high immunoreactivity for CD34 and p63. CD34 is a sensitive marker of the vascular endothelium and strong CD34 staining is usually associated with tumor relapse or metastasis (14). p63 is a p53 homolog that is expressed in a variety of normal epithelial tissues and epithelial malignancies. It has been documented that p63 serves as a sensitive and specific myoepithelial marker in benign and malignant breast lesions (15). Taken together, these pathological results confirm a malignant nature of a recurrent tumor.

Cytokeratin is a surface marker expressed on epithelial cells, while vimentin is a member of the intermediate filament family and is expressed in mesenchymal cells. In the present case, the recurrent tumors prior to and following total mastectomy consistently demonstrated strong immunostaining for vimentin. By contrast, cytokeratin immunoreactivity was only observed in the recurrent tumor following surgery. The concurrent positive immunostaining for cytokeratin and vimentin indicated mixed epithelial- and mesenchymal-type tumor cells in the malignant neoplasm following surgery. The acquired expression of cytokeratin may indicate its critical role in the malignant transformation of IMT of the breast. Cytokeratin expression has also been found to be associated with aggressive potential and a poor prognosis in numerous malignancies, including breast (16) and laryngeal cancer (17), which further validates this hypothesis.

Surgery remains the first-line treatment option for IMT. A pre-operative evaluation, aiming to differentiate between benign and malignant lesions, is critical for selecting a surgical modality. There is currently no reliable biomarker for predicting the nature of IMT. However, the results of the present case indicated that rapid tumor development and a high Ki-67 labeling index may be indicators for extended radical mastectomy. Complete surgical excision is critical for achieving a good prognosis in patients with IMT. Additionally, long-term follow-up is mandatory, as certain cases may have the potential for malignant transformation.

In conclusion, in the present case of IMT of the breast, malignant transformation to a metaplastic carcinoma of the spindle-cell type was observed following surgical intervention. Such malignant progression may be ascribed to incomplete initial surgical resection due to misdiagnosis as a benign lesion. Therefore, differentiation between aggressive and non-aggressive forms of IMT is critical in the choice of surgical approaches. Rapid tumor growth and a high Ki-67 labeling index may indicate a high risk of recurrence.
